# Predicting the Fracture Toughness of Human Cancellous Bone in Fractured Neck of Femur Patients Using Bone Volume and Micro-Architecture

**DOI:** 10.3390/life14040467

**Published:** 2024-04-03

**Authors:** George J. Adams, Richard B. Cook, Michael Gibson, Peter Zioupos

**Affiliations:** 1 Cranfield Forensic Institute, Cranfield University, Cranfield MK43 0AL, UK; g.adams1992@gmail.com (G.J.A.);; 2 nCATS, School of Engineering Science, University of Southampton, Southampton SO17 1BJ, UK; r.b.cook@soton.ac.uk; 3 Biomedical Engineering Research Group, School of Engineering, University of Hull, Kingston upon Hull HU6 7RX, UK

**Keywords:** cancellous bone, fracture toughness, BV/TV, micro-architecture, crack growth, microCT, multi-regression predictions

## Abstract

The current protocol used to determine if an individual is osteoporotic relies on assessment of the individual’s bone mineral density (BMD), which allows clinicians to judge the condition of a patient with respect to their peers. This, in essence, evaluates a person’s fracture risk, because BMD is a good surrogate measure for strength and stiffness. In recent studies, the authors were the first to produce fracture toughness (FT) data from osteoporotic (OP) and osteoarthritic (OA) patients, by using a testing technique which basically analyzes the prerequisite stress conditions for the onset of growth of a major crack through cancellous bone tissue. FT depends mainly on bone quantity (BV/TV, bone volume/tissue volume), but also on bone micro-architecture (mArch), the inner trabecular design of the bone. The working research hypothesis of the present study is that mArch offers added prediction power to BV/TV in determining FT parameters. Consequently, our aim was to investigate the use of predictive models for fracture toughness and also to investigate if there are any significant differences between the models produced from samples loaded across (A_C_, transverse to) the main trabecular orientation and along (A_L_, in parallel) the trabeculae. In multilinear regression analysis, we found that the strength of the relationships varied for a crack growing in these two orthogonal directions. Adding mArch variables in the Ac direction helped to increase the R^2^ to 0.798. However, in the A_L_ direction, adding the mArch parameters did not add any predictive power to using BV/TV alone; BV/TV on its own could produce R^2^ = 0.730. The present results also imply that the anisotropic layout of the trabeculae makes it more difficult for a major crack to grow transversely across them. Cancellous bone models and remodels itself in a certain way to resist fracture in a specific direction, and thus, we should be mindful that architectural quality as well as bone quantity are needed to understand the resistance to fracture.

## 1. Introduction

Osteoporosis (OP) is a degenerative bone condition that is thought to be responsible for 8.9 million fractures per year [1]. It is estimated that one in two women and one in five men over the age of 50 will suffer a fragility fracture, which is defined as a fracture caused by a fall from standing height or less. These fractures are typically associated with or attributed to osteoporosis or osteopenia. In the UK alone there were approximately 527,000 new fragility fractures in 2019, estimated to increase by 26.2% to 665,000 in 2034, the cost similarly rising constantly by GBP~100 million/year from its present level of GBP~5.0 billion/year [2]. At present, OP is defined as having a bone mass 2.5 standard deviations below the young adult reference mean [3]. Another prevalent condition that affects bone tissue is osteoarthritis. Osteoarthritis is normally considered only for its impact on the articular cartilage of the synovial joints; the knock-on effects of the compromised joints causes structural changes to occur in the subchondral bone [4]. Osteoarthritis affects 8.75 million people in the UK, and it is estimated that 33% of the population over the age of 45 have sought treatment for osteoarthritis. The joints most affected by the condition are the knee and hip affecting 4.7 and 2.46 million people, respectively.

Current protocol in determining a patient’s fracture risk and whether they are osteoporotic is based on dual energy X-ray absorptiometry (DEXA). This assessment using DEXA gives an indication of the patient’s bone mineral density (BMD) which is the product of both the porosity and density of the mineralized bone tissue; this is usually taken at the hip [5]. The DEXA results are assessed using the fracture risk assessment tool as recommended by the World Health Organization. While this provides valuable data on an individual’s fracture risk, advancements in medical imaging technology allow for development of more robust and accurate risk assessment tools [6].

The primary role of bone in the body is as a structural material and the cancellous regions can be considered as a cellular solid [7,8,9,10,11]. As such, the mechanical properties of cancellous bone are impacted by the base material properties of the structure and the micro-architecture of the structure. All variations in the micro-structural properties of the tissue, from the quantity of bone tissue to the orientation of individual trabecular architecture, will impact the resultant mechanical properties of the tissue. The current DEXA protocol, however, fails to consider the architecture of the individual trabeculae. The most common mechanical property that is investigated is the compressive strength of the bone tissue, which fails to consider the ability of the tissue to resist fracture, an extremely important consideration when assessing the ability of bone to carry out its daily tasks, specifically its ability not to fracture under load. This has been considered by a previous study [11], in which the fracture toughness of discs and beams of cancellous bone were measured, conforming to ASTM standards.

Multiple regression represents an advancement beyond traditional linear regression, because it allows the utilization of multiple predictors to estimate the value of a variable based on the values of two or more predictors. It also assesses the collective impact of multiple predictors on determining the outcome, providing a comprehensive understanding of the overall fit. It is a tool rarely used to predict the mechanical response of bone based on its architecture and has never before been used to predict the fracture toughness of bone in these terms. The authors recognize that in the application of multiple linear regression, the resultant models are not prescriptive of the underlying mechanisms but rather a descriptive method to ascertain the relationships within the sample set.

In a series of recent studies, we have demonstrated the importance and impact of changes in the micro-architecture and material properties in cancellous bone mechanics [5,12,13,14,15,16,17], and these will provide a basis for the work that is presented here. In this study, we have the two following primary objectives: (a) investigate the use of predictive models to help in the prediction of fracture toughness, and (b) investigate if there are any significant differences between the models produced from samples loaded in the across (A_C_) and along (A_L_) loading configurations.

## 2. Materials and Methods

### 2.1. Bone Specimens

A sample set of femoral heads were collected from 37 osteoporotic (OP) and 8 osteoarthritic (OA) patients who had received a total hip replacement surgery due to suffering fragility fractures at the femoral neck (OP) or elective surgery for arthroplasty (OA). During the surgery, specialist surgeons were able to remove the femoral heads intact. The femoral head was used in this study due to the increased availability of tissue at the site compared to the femoral neck, where fracture typically occurs, whilst being physically close to the femoral neck. This cohort allowed for the collection of a number of samples, covering a broad range of porosity values, with OP covering the lower end of the spectrum and OA the normal and higher end of porosities. A good overlap was shown between the two groups. Population characteristics are provided in Table 1. Following removal, all samples were kept at −20 °C prior to sample preparation. Ethical approval for the collection and use of these specimens was provided by Gloucestershire NHS trust REC (acknowledgments).

### 2.2. Specimen Preparation

**Figure 1:**
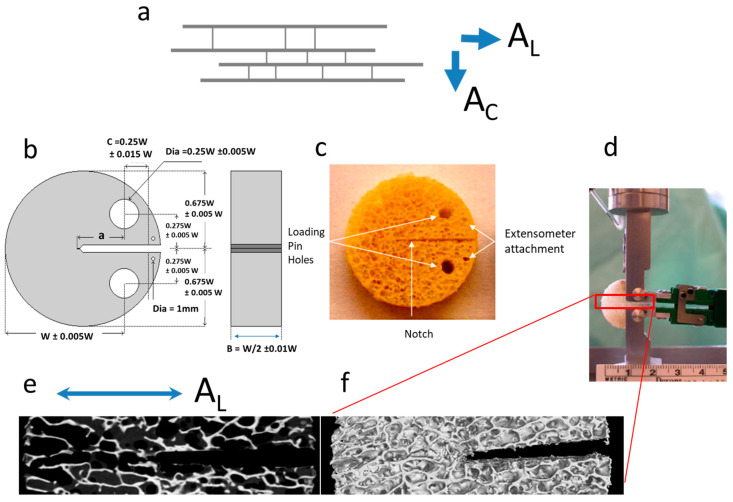
(**a**) Cancellous bone samples have their trabeculae oriented, in the majority of cases, along the direction of the principal axes of stress and thus they are anisotropic. A propagating crack will find its way either advancing between the trabeculae (along direction A_L_) or transversely across the trabeculae (across direction A_C_); (**b**) design template for preparing the disk-shaped fracture toughness specimens [4]; (**c**) example of a cancellous bone sample prepared according to the template; (**d**) sample mounted on the grip, under continuous physiological fluid irrigation and with extensometer attached at the mouth of the crack; (**e**,**f**) μ-CT scanned section for the portion of the sample along the path of the growing crack (magnified 5.25), this particular sample is an A_L_ oriented one; where (**e**) single slice along a sagittal plane through the sample; and (**f**) a 3D rendering of the sample surface using all the μ-CT data.

Specimen preparation (including sectioning from the femoral head and cleaning) has previously been described in detail [4,11]. Single Edge Notched Disc (SEND) samples were prepared to conform to an adjusted ASTM standard E399-90, in order to assess the necessary stress conditions to instigate crack growth from a man-made notch (Figure 1). Samples were divided into two subsets; with 34 samples orientated along (A_L_) the primary direction of the trabecular and 27 orientated across (A_C_) the primary orientation of the trabecular structure. Due to it being a cellular solid comprising struts and beams, directionality is a natural feature of cancellous bone. When the struts and beams are not isotropically laid out, they result in an anisotropic structure, where directions such as along and across the majority of the beams can be discerned. In a few femoral heads, samples were produced in both A_L_ and A_C_ directions. All specimens were stored at −20 °C following a defatting process detailed in [4,11]. The sectioning was performed by using a metallurgical saw (Struers^®^ Accutom-2, Rotherham, UK). The samples were then sanded and polished by using progressively finer grades of carbide paper (400–2500 grit) to the dimensions required for material testing. Specimens were manufactured in the shape of discs, diameter 20 mm and thickness 7.5 mm, for mechanical material testing as SEND. Sample preparation was performed under constant water irrigation, to prevent the production of micro-cracks or other damage to the specimens.

### 2.3. Micro-Computed Tomography

The samples’ micro-architecture was imaged using cone beam micro-computed tomography (μ-CT, μ-CBCT). Each sample was imaged using a Nikon CT H225 (X-Tek Systems Ltd., Tring, Hertfordshire, UK) cone beam μ-CT (μ-CBCT) scanner. Samples were imaged at 50 kV and 65 μA with a 1000 ms exposure. The resultant voxel size of the scan was ~24 μm. All scans were manually reconstructed using CT Pro 3D. During reconstruction, conditions were optimized to reduce beam hardening and noise, and the noise and beam hardening corrections were standardized across all the samples to ensure that the results were comparable. Image analysis and visualization were carried out using VG Studio Max 2.2 (Volume Graphics GmbH, Heidelberg, Germany). Firstly, the samples’ structural properties were determined, and these parameters included the following: trabecular thickness (TbTh), spacing (TbSp) and number (TbN), surface area (BS), material volume (BV), and total volume (TV). The density of the samples was also determined using a QRM MicroCT-HA (QRM GmbH, Möhrendorf, Germany) calibration phantom. This uses hydroxyapatite of different known concentrations to produce a calibration curve of gray value versus density. Using this calibration, the density of the samples can be determined, which is often referred to as tissue mineral density or material density (D_mat_). Following the determination of the D_mat_ from the average gray value, the apparent density (D_app_) of each sample was determined using Equation (1). The D_app_ is often referred to as bone mineral density.

D_app_ = D_mat_ × BV/TV (1)

In this article, Dmat and Dapp are used to indicate that they have been calculated on a volumetric basis using μ-CT data. This is important to distinguish, as bone mineral density measured in DEXA is an areal representation of this bone mArch parameter. BoneJ© [http://bonej.org/; http://rsbweb.nih.gov/ij/] (30 June 2018) was employed at a second stage to calculate additional micro-architectural parameters such as structure model index (SMI), degree of anisotropy (DA), connectivity density (Conn. D) and Euler characteristic (Euler ch.) [18].

### 2.4. Mechanical Testing

The SEND samples were mechanically characterized, as in previous papers, for fracture toughness using a linear elastic FM approach [4,11]. The Kc (critical stress intensity factor) values were derived for the load at a point where the man-made notch started growing (following extensive yielding and bending of the trabeculae ahead of the notch), caused by snapping of one or more trabeculae in the first instance. The deformation was measured by a miniature extensometer (Model 3442-006M-050ST, DWE Scientific Ltd., Brackley, UK) attached at the mouth of the notch. The dimensions and other restrictions that were followed complied with the usual material testing standards such as ASTM E399-90, as reported in [11]. The mechanical testing was undertaken using a DARTEC^®^ Series HC25 (Zwick Roell, Worcester, UK) materials testing machine driven by a 9610 series controller unit and operated using Workshop 96© software. The load was monitored using a 500 N load cell (RDP Electronics Ltd., Wolverhampton, UK) whilst the gauge length of the crack mouth opening displacement measured by the extensometer was 6 mm. The loading rate during fracture toughness testing was 0.05 mm s^−1^ (3 mm min^−1^), with data acquisition at a capture rate of 1000 points per minute. Unlike the more common compression studies, which have tested cancellous bone in cylinders or cubes, these tests were the first ever to attempt a quantification of the necessary loading conditions that would allow a crack to start growing from stability into an unstable fracture mode. In this respect, the mechanical data offer a novel and invaluable way of assessing the structural integrity and loading ability of these samples, in a way that resembles the conditions in FNF situations in a more biofidelic manner [11].

### 2.5. Statistical Analysis

Statistical analysis was carried out in two steps. Firstly, comparisons were made among the multiple subgroups within the cohort, such as the A_L_ and A_C_ loading configurations, and the OP and OA groups. Pearson’s correlation coefficients were used throughout the study. In the second stage of statistical analysis, multiple linear regressions were employed to explore to what degree BV/TV and the full set of mArch parameters are able to predict Kc. The multiple regression was a stepwise selection in MINITAB© (v.17). The stepwise selection combined forward selection and backward elimination, where added variables were deleted if their contribution to the model is not determined to be significant. It must be considered that the values for trabecular thickness (TbTh) and trabecular number (TbN) are calculated interdependently; therefore, in the development of statistical models, inclusion of both has been avoided.

## 3. Results

Table 2 shows a descriptive and statistical comparison of the A_L_ and A_C_ as separate groups, as well as the average parameters collected for the entire cohort. Values measured between the groups were not statistically significantly different, with the exception of DA, which may be an artefact of the cutting and selection process. Even with this consideration in mind, it shows that the differences between the subsequent correlations and regression analysis is due to the contribution of the parameters to the loading in the specified direction. A comparison of the morphological data between males and females and with other studies was reported in a previous study [5].

As well as considering the different loading conditions, differences between the OP and OA groups and the relationship they have with fracture toughness are also considered. Therefore, the relationships between the architectural properties of OP and OA bone and their corresponding fracture toughness are presented (Table 3). Within the OP sample set, Kc had a higher correlation with trabecular spacing than observed in the OA group, whereas BV/TV and TbN correlations in the OA groups were much higher than in the OP. There was a consistency in parameters that correlated significantly between the OP and OA groups, except for connectivity density (Conn. D), which was found to be significant (*p* < 0.05) in the OA group but not in the OP group. Table 3 also includes a comparison of how the morphological data collected here compares to previous studies.

### 3.1. Micro-Architecture

Table 4 shows the correlations between the micro-architectural parameters and fracture toughness, with R^2^ and *p*-values given, in the A_L_ and A_C_ groups as well as in the combined groups. The parameter with the highest R^2^ value in the A_L_ and combined groups was BV/TV whilst in the A_C_ group it was the TbN and BS/TV. Most of the parameters measured were found to impact upon fracture toughness, except for DA across the entire cohort and Conn. D in the A_C_ loading group. In Table 5, the correlations within the OP and OA separate groups are shown. When considering the entire cohort, the BV/TV had the highest R^2^ value in both groups. The DA and Conn. D did not correlate significantly in either group. Additionally, the Dmat was seen not to be significant with the OP/OA separation.

### 3.2. Regression Analysis

**Figure 2:**
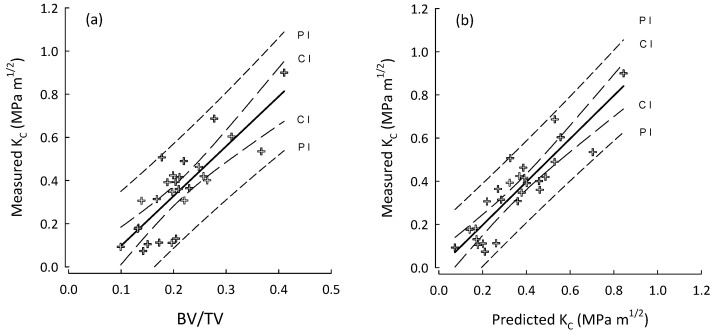
Plots for the A_C_ group (crosses) of (**a**) BV/TV vs. measured fracture toughness (K_C_); and (**b**) best stepwise regression model (step-3, Table 7) for predicted K_C_ vs. measured K_C_. Regression lines with their 95% confidence (CI) and prediction (PI) intervals.

**Figure 3:**
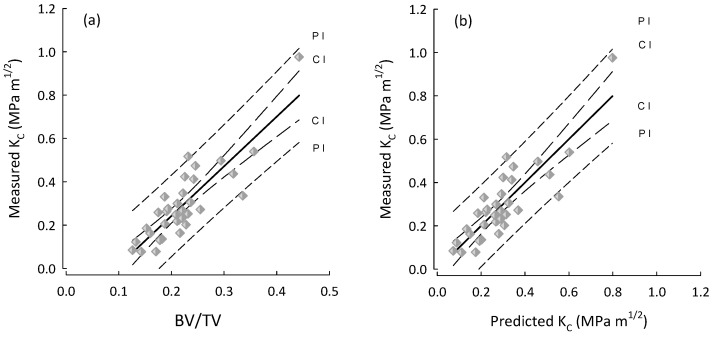
Plots for the A_L_ group (diamonds) of (**a**) BV/TV vs. measured fracture toughness (K_C_); and (**b**) predicted K_C_ vs. measured K_C_. The performance is the same because the best predictor for Kc (A_L_) is in fact BV/TV. Regression lines with their 95% confidence (CI) and prediction (PI) intervals.

**Figure 4:**
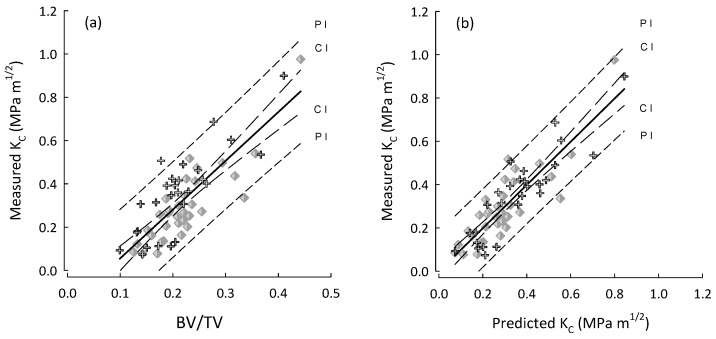
Plots for the entire cohort (OP and OA) and for both A_C_ (crosses), A_L_ (diamonds) in the following: (**a**) BV/TV vs. measured fracture toughness (K_C_); and (**b**) predicted K_C_ vs. measured K_C_ by using the best stepwise regression model (step-4, Table 8) for the entire cohort. Regression lines with their 95% confidence (CI) and prediction (PI) intervals.

For multiple regressions, BV/TV was taken to be the base predictor as it was consistently the parameter that correlated highest with fracture toughness. Additionally, BV/TV is very closely linked to the metric currently used in the assessment of OP, as results from DEXA are mostly influenced by the quantity of bone rather than the density of the material itself [5]. The performance of predictions produced by multiple regressions are shown in Figure 2, Figure 3 and Figure 4. For A_C_ samples (Figure 2) the inclusion of additional mArch parameters added to the predictive power of BV/TV alone. However, as shown in Table 6, stepwise regression was unable to identify any additional mArch parameters that could significantly improve the R^2^ value for the A_L_ group beyond BV/TV alone. The R^2^ values for the A_L_ group for BV/TV and stepwise best fits were the same. The final best fit for the A_C_ group is given in Table 7 and for the entire cohort in Table 8. 

The best models utilizing as many as possible variants, at a *p*-value < 0.05, were step-3 (Table 7) for the A_C_ group, and step-4 (Table 8) for the entire cohort. There are two technical aspects of applying the stepwise regressions that are worth noting: (i) to add or subtract a parameter to the model, the *p*-value was set to 0.15; and (ii) applying multiple regressions to the entire cohort adhered to the ‘rule of ten’ which suggests a minimum of ten samples for every predictor in the model. However, in the A_L_ groups model this was not maintained. Whilst it has been suggested that this is not necessary, maintaining a high number of predictors to samples is advantageous and helps reduce the effects of over fitting [35].

## 4. Discussion

The research presented in this article outlines the fundamental relationships between the fracture toughness of cancellous bone and the material quality factors measured by μ-CT, and implements the use of statistical models to predict the mechanical properties of the samples. The collection of samples, which have been used in previous studies, are unique in that they are the only instance of measuring cancellous fracture toughness considering the start of growth of a major crack [4,11], as opposed to the total work under the load/deformation curve [36]. Previous μ-CT research on this cohort has investigated the micro-architecture and material quality whilst looking at differences between the male and female samples in the cohort, and treated the samples loaded in different configurations (A_L_ and A_C_) indiscriminately [5]. Here, we have taken the opposite approach and treated the male/female samples indiscriminately and separated the A_L_ and A_C_ loading configurations. This research also has the inclusion of OA samples which represent perhaps the opposite of OP, in that the effects of OA tend to lead to a thickening of the subchondral bone. The use of μ-CT imaging presents an opportunity to assess the skeleton not currently found across the array of medical machines available. Current OP diagnosis by DEXA assesses the BMD which is a representation of the density of cancellous architecture and the material density of the bone itself. Medical CT scanners can also be used to assess the structure of the skeleton; however, the voxel size and image resolution currently obtainable from these systems is nowhere near as great as that which can be achieved in μ-CT. Therefore, all data and its associated methodology presented here represent what could potentially be assessed in the future, and are precursors to future non-invasive assessment of bone fracture toughness in diagnostic clinics, if we could only develop the ability to assess these same characteristics in vivo.

As previously mentioned, there is a real danger of over fitting data in a multiple regression analysis, which would produce models that claim to predict better than they are capable of. Here, we have taken every care to include the fewest number of predictors and to ensure that the predictors are independent of each other. In bone, however, this is very difficult due to the dependence of parameters on other physical characteristics, including both the obvious links between BV/TV and apparent density, and the less apparent links between the material density and the BV/TV [34]. Multiple regression analysis was not carried out in the OP and OA subgroups due to a very small samples size of the OA group. The SMI values reported in this study were negative; this is due to the samples containing a significant number of concave surfaces. In the SMI calculation, it is assumed that the number of concave surfaces is negligible [37]. Therefore, SMI was excluded from the multiple regression models due to lack of suitability but was included to demonstrate that the number of concave surfaces in cancellous bone are significant.

The comparison of OP and OA subgroups has supported the notion that OP leads to the loss of bone, shown by the significant differences between the BV/TV of the two groups (*p* < 0.01). The average BV/TV of the OA group is still within the range previously reported in the literature (Table 3), suggesting that in the OA condition, there is no extreme deposition of new bone tissue within the cancellous regions. The measured BS/BV and calculated BS/TV are within the literature ranges for both OP and OA groups. The differences between BS/BV for the OP and OA groups suggest that there are more surfaces available within the OP groups, which is consistent with the notion that remodeling is a surface effect [29,38]. Therefore, greater rates of remodeling could lead to a loss of bone which is typically associated with OP [39,40]. The trabecular number and thickness are higher in the OA group, which is typically consistent with the increased BV/TV and consistent with increases in mechanical strength [9,11,38,41]. There were no significant differences in the morphology measured between the A_L_ and A_C_ groups suggesting that any differences between correlations with the architectural parameters and any differences in the multiple regression models produced are due to the contributions of the individual parameters in the different loading directions.

When looking at the entire sample set, BV/TV was seen to have the highest correlation with fracture toughness, enforcing the assertion that the quantity of bone is the biggest contributor to bone strength. However, in the division of the A_L_ and A_C_ subgroups this only held true for the A_L_ group, whilst in the A_C_ group the TbN was seen to have the highest correlation. This suggests that a denser trabecular packing may have a bigger impact on cancellous bones’ resistance to fracture in the A_C_ loading configuration than in the A_L_. The significant Dmat correlation across all the groups suggests that the material composition of the bone tissue plays an important role in the ability of the tissue to resist fracture, which supports previously found differences between the physio-chemistry of normal and OP bone tissue [16]. However, the effect of Dmat is clearly not as important as the structural properties of the tissue as evidenced by the much lower R^2^ values. Between the OP and OA subgroups, the parameters that impacted on fracture toughness followed the same trends, except for connectivity density (Conn. D), which was a significant contributor in the OA group but not the OP. This is perhaps due to the connectivity density being significantly higher in the OA group.

### Multiple Regressions

Using multiple linear regressions, we were able to demonstrate that multiple morphological parameters impact upon the fracture toughness of bone when loaded in the A_C_ direction or when loading direction is not considered. By accounting for these parameters within the model, it is possible to better predict the fracture toughness of bone than by consideration of multiple parameters. However, in the A_L_ group, the use of multiple regression was unable to identify any parameter that would significantly improve the model. This has very profound implications on the understanding of bone fracture toughness and suggests that in the A_L_ loading direction, the only parameter that resists fracture is the quantity of bone available, and that other parameters such as the average thickness of trabeculae do not develop in such a way to resist fracture. In the A_C_ direction, however, other parameters had a significant effect on the ability of material to resist fracture. This is consistent with basic underpinning mechanisms of bone remodeling suggested by Wolff, whereby bone is responsive and adapts to the loads applied to it. The samples in the A_C_ groups are orientated across the primary direction of loading in the hip, so the bone will have adapted to resist fracture in this direction and as such, this adaptation has led to reorientation of the trabeculae to achieve this. The A_L_ group were orientated perpendicular to the primary loading of bone and as such, the trabecular structure has not adapted in micro-orientation to resist fracture.

The two primary aims of this study have been addressed as follows: (a) we have shown that across the entire cohort, consideration of multiple morphological parameters can help produce models that can inform on bone quality and can perhaps be used to predict fracture toughness with further development; and (b) separation of the models produced between the A_L_ and A_C_ groups was found to be revealing, in that for the A_L_ group, no additional parameter was seen to improve the predictive ability over and above the use of BV/TV. This is incredibly surprising and has implications on our comprehension of how bone at the hip remodels to help resist fractures. To conclude, the use of multiple regressions represents a real opportunity to develop models to predict the likelihood of a patient’s fracture using bone micro-architecture, and there is a clear case for investigation into the remodeling of bone at the hip.

## 5. Conclusions

This study has considered the impact of the micro-architecture of cancellous bone on the fracture toughness of the tissue. We have been able to use a relatively large cohort of samples collected from patients undergoing hip replacement surgery and determined to be either osteoporotic or osteoarthritic. The findings support the currently used DEXA model, whereby a significantly reduced bone mass leads to a reduction in the mechanical competency of the tissue. It has additionally supported previous reports that multiple structural parameters such as TbTh, TbSp, TbN, and BS/BV also contribute significantly to the fracture toughness. We also employed the use of a statistical tool, multiple regression analysis, to demonstrate that the combination of multiple structural parameters can lead to an improved model of fracture toughness that may provide a basis to predict the fracture risk of a patient. The use of multiple regressions also highlighted that in the A_L_ loading condition, the quantity of bone is the biggest contributor to fracture toughness and that the inclusion of additional parameters did not significantly improve the predictive power. The same cannot be said for the A_C_ group, which showed a marked improvement with the addition of multiple parameters. This further proves Wolff’s law, or at least the principle, that bone truly remodels to its loading, and in this case, to resist fracture at the hip. The use of multiple regression is not without its limitations; in this study, from a statistical perspective, the sample size is relatively small; however, from a study on human bone samples perspective, it can be considered relatively large. Further work is required to investigate if these architectural parameters can be included alongside the currently collected BMD to improve the prediction of patients’ fracture risk.

## Figures and Tables

**Table 1:** Anthropometric and demographic data of OP and OA groups.

	OP	OA
Donors	37	8
Male/Female	7/30	5/3
Number of specimens	60	19
Age range (years)	59–96	53–76
Age mean (years)	82.3 (SD = 6.8)	66 (SD = 7.3)
Weight range (kg)	41.3–82.6	68–108
Weight mean (kg)	64.2 (SD = 10.5)	84.5 (SD = 12.96)
Height range (m)	1.55–1.80	1.65–1.83
Height mean (m)	1.67 (SD = 0.08)	1.76 (SD = 0.074)

SD: standard deviation.

**Table 2:** Average micro-architecture properties for samples loaded as A_L_ and A_C_. Standard deviation (SD) and *p*-values denoting significant difference (*p* < 0.05) between the two groups are included. (Abbreviations for the parameters are explained in the micro-computer tomography section in Materials and Methods.)

	All Samples		A_L_ (N = 34)		A_C_ (N = 27)		
Parameter	Mean	SD	Mean	SD	Mean	SD	*p*-Values
BV/TV	0.21	0.079	0.23	0.09	0.202	0.073	0.14
BS/BV	14.53	2.62	13.99	2.43	15.00	2.72	0.09
BS/TV	2.94	0.57	3.03	0.62	2.86	0.51	0.18
TbTh	0.14	0.029	0.15	0.03	0.14	0.03	0.14
TbN	1.44	0.34	1.50	0.29	1.38	0.38	0.10
Tbsp	0.60	0.24	0.54	0.14	0.66	0.29	0.06
DA	2.37	0.68	2.55	0.81	2.21	0.49	**0.02**
Conn. D	2.18	1.14	2.20	1.25	2.16	1.04	0.13
SMI	−5.87	4.65	−4.92	3.73	−6.70	5.24	0.10
D_mat_	1.79	0.08	1.81	0.07	1.77	0.36	0.88
D_app_	0.39	0.16	0.41	0.17	0.08	0.14	0.09

**Table 3:** Average micro-architecture properties for the OP and OA groups. Standard deviation (SD) and *p*-values denoting significant difference (*p* < 0.05) between the two groups are included. Micro-architecture values from other studies are also provided for comparison (modified from [4]) (Abbreviations for the parameters are explained in the micro-computer tomography section in Materials and Methods).

	OP		OA				
Parameter	Mean	SD	Mean	SD	*p*-Values	Range in the Literature	References
Kc	0.29	0.14	0.42	0.28	0.02	-	-
BV/TV	0.20	0.05	0.27	0.094	<0.01	0.07–0.30	[19,20,21,22,23,24,25]
BS/BV (mm^−1^)	14.62	2.37	12.79	2.25	0.01	8.70–22.5	[25,26,27]
BS/TV (mm^−1^)	2.86	0.47	3.33	0.75	<0.01	0.59–5.00	[28,29]
TbTh (mm^−1^)	0.14	0.02	0.16	0.03	<0.01	0.09–0.25	[20,21,22,24,25,30]
TbN (mm^−1^)	1.46	0.25	1.63	0.34	0.04	0.76–2.52	[22,24,25,29,31]
Tbsp (mm^−1^)	0.57	0.13	0.50	0.16	0.15	0.30–1.22	[21,22,24,25,31]
DA	2.41	0.62	2.25	0.81	0.16	1.73–2.00	[21,24]
Conn. D	1.83	0.88	3.36	0.94	<0.01	1.96–5.62	[32]
SMI	−6.15	3.48	−1.86	2.89	<0.01	0.50–2.61	[21,24,27,30]
D_mat_ (g cm^−3^)	1.80	0.09	1.80	0.05	0.74	1.40–2.00	[9,11,33,34]
D_app_ (g cm^−3^)	0.36	0.09	0.50	0.18	<0.01	0.12–0.37	[23,25]

**Table 4:** R^2^ and *p*-values for correlations between architectural parameters and fracture toughness (bold typeface for *p* < 0.05). (Abbreviations for the parameters are explained in the micro-computer tomography section in Materials and Methods).

	All		A_L_		A_C_	
Parameter	R^2^	*p*-Value	R^2^	*p*-value	R^2^	*p*-Value
BV/TV	**0.66**	<0.01	**0.74**	<0.01	**0.67**	<0.01
BS/BV	**0.25**	<0.01	**0.25**	<0.01	**0.35**	<0.01
BS/TV	**0.57**	<0.01	**0.51**	<0.01	**0.72**	<0.01
TbTh	**0.34**	<0.01	**0.37**	0.025	**0.39**	<0.01
TbN	**0.56**	<0.01	**0.51**	<0.01	**0.72**	<0.01
Tbsp	**0.40**	<0.01	**0.49**	<0.01	**0.41**	<0.01
DA	0.00	0.74	0.01	0.60	0.10	0.11
Conn. D	**0.16**	<0.01	**0.26**	<0.01	0.09	0.13
D_mat_	**0.11**	0.01	**0.13**	0.04	**0.18**	0.025
D_app_	**0.64**	<0.01	**0.73**	<0.01	**0.65**	<0.01

**Table 5:** R^2^ and *p*-values for correlations between architectural parameters and fracture toughness with OP and OA groups separated (bold typeface for *p* < 0.05). (Abbreviations for the parameters are explained in the micro-computer tomography section in Materials and Methods).

	OP		OA	
Parameter	R^2^	*p*-Value	R^2^	*p*-Value
BV/TV	**0.58**	<0.01	**0.69**	<0.01
BS/BV	**0.17**	<0.01	**0.37**	0.02
BS/TV	**0.52**	<0.01	**0.56**	<0.01
TbTh	**0.21**	<0.01	**0.40**	0.02
TbN	**0.44**	<0.01	**0.71**	<0.01
Tbsp	**0.48**	<0.01	**0.29**	0.05
DA	0.01	0.49	0.01	0.72
Conn. D	0.03	0.28	**0.28**	0.05
D_mat_	0.11	0.22	0.23	0.08
D_app_	**0.54**	<0.01	**0.68**	<0.01

**Table 6:** R^2^, Adjusted R^2^ (bold) and Significance-F multiple linear regressions for all samples as well as a separation of the A_L_ and A_C_ loading groups.

	All			A_L_			A_C_		
	R^2^	Adj-R^2^	Signif-F	R^2^	Adj-R^2^	Signif-F	R^2^	Adj-R^2^	Signif-F
BV/TV	0.670	**0.656**	<0.001	0.738	**0.730**	<0.001	0.674	**0.661**	<0.001
Stepwise selection	0.759	**0.741**	<0.001	0.738	**0.730**	<0.001	0.798	**0.771**	<0.001

**Table 7:** Stepwise regression steps for the A_C_ group using BV/TV as the base predictor with the addition of degree of anisotropy (DA), trabecular thickness (TbTh), connectivity density (Conn. D), and trabecular spacing (Tbsp) (Alpha to add 0.05), the final step with all predictors being significant (*p* < 0.05) is given in bold. (Abbreviations for the parameters are explained in the micro-computer tomography section in Materials and Methods).

	Step				
	1	2	3	4	5
Constant	−0.133	−0.436	**−0.125**	9.565 × 10^−6^	−0.262
BV/TV	2.31	2.26	**3.49**	3.97	4.74
T-value	7.18	7.77	**6.21**	6.46	6.19
*p*-value	<0.001	<0.001	**<0.001**	<0.001	<0.001
DA		0.60	**0.62**	0.50	0.53
T-value		2.57	**2.93**	2.27	2.47
*p*-value		0.017	**0.008**	0.033	0.022
TbTh			**−4.1**	−4.7	−5.4
T-value			**−2.48**	−2.85	−3.26
*p*-value			**0.021**	0.009	0.004
Conn. D				−0.040	−0.044
T-value				−1.65	−1.85
*p*-value				0.114	0.079
Tbsp					0.32
T-value					1.59
*p*-value					0.126
S	0.115	0.104	**0.094**	0.091	0.088
R^2^	0.673	0.744	**0.790**	0.820	0.840
R^2^ (adjusted)	0.660	0.722	**0.771**	0.787	0.801

**Table 8:** Stepwise regression steps for the entire cohort using BV/TV as the base predictor with the addition TbN, Tbsp, BS/TV, and DA (Alpha to add 0.15), the final step with all predictors being significant (*p* < 0.05) is given in bold. (Abbreviations for the parameters are explained in the micro-computer tomography section in Materials and Methods).

	Step				
	1	2	3	4	5
Constant	−0.1712	−0.3650	−1.0018	**−1.0062**	−1.1267
BV/TV	2.27	1.37	1.60	**1.81**	1.74
T-value	10.83	3.48	4.09	**4.71**	4.54
*p*-value	<0.001	<0.001	<0.001	**<0.001**	<0.001
TbN		0.262	0.458	**0.983**	1.006
T-value		2.64	3.65	**3.96**	4.10
*p*-value		0.011	0.001	**<0.001**	<0.001
Tbsp			0.53	**0.54**	0.55
T-value			2.40	**2.53**	2.62
*p*-value			0.020	**0.014**	0.011
BS/TV				**−0.28**	−0.27
T-value				**−2.42**	−2.36
*p*-value				**0.019**	0.022
Degree of Anisotropy					0.028
T-value					1.54
*p*-value					0.129
S	0.109	0.104	0.099	**0.096**	0.095
R^2^	0.669	0.705	0.733	**0.758**	0.769
R^2^ (adjusted)	0.663	0.695	0.718	**0.741**	0.747

## Data Availability

Data for this manuscript when the article is in print will be available through the Cranfield University CORD data depository and preservation system at https://cranfield.figshare.com, or through the corresponding author p.zioupos@hull.ac.uk.
